# Comparative study of OMNIgene®•SPUTUM reagent versus cold-chain for the transportation of sputum samples to GeneXpert®MTB/RIF testing sites in Malawi

**DOI:** 10.1186/s12879-019-4070-8

**Published:** 2019-05-16

**Authors:** Wondwossen A. Alemayehu, S. Neri, S. Dalebout, R. Nalikungwi, A. Trusov, E. Ahmed, A. Dimba, A. Weirich, P. S. Curry, C. D. Kelly-Cirino

**Affiliations:** 1 Project HOPE, United States and Africa, PO Box 13395, Addis Ababa, Ethiopia; 2 Ministry of Health, National TB Program, Lilongwe, Malawi; 3 DNA Genotek, Ottawa, Ontario Canada

**Keywords:** OMNIgene, Sputum, GeneXpert, *Mycobacterium tuberculosis*, Preservation, Specimen transport medium, Molecular detection

## Abstract

**Background:**

The study was conducted in a remote sputum sample collection sites and GeneXpert® MTB/RIF testing centers to detect *Mycobacterium tuberculosis* in Malawi. The main purpose of the study was to evaluate whether sputum samples stored and transported with OMNIgene®•SPUTUM (OM-S) medium perform comparably to the routine cold-chain stored and transported samples for GeneXpert testing to detect *Mycobacterium tuberculosis*.

**Methods:**

Two sputum samples from each of 362 tuberculosis suspects were randomly assigned to the OMNIgene treated (OM-S group) or the standard-of-care group (SOC; transported via cold chain). All specimens were tested at regional GeneXpert testing sites using the expectorated (raw) sputum protocol. Demographic, clinical, transport/storage and Xpert data were recorded for each specimen pair. Agreement between the SOC and OM-S groups’ Xpert results was evaluated using *Cohen’s kappa* analysis.

**Results:**

Mean patient age was 42.3 years (range 2–79 years), 77% of patients were female, and 80% were HIV-positive. Mean transport/storage time was 6.7 days (range, 0–29 days). The rates of MTB positivity for the OM-S and SOC groups were comparable (11.8 and 11.2%, respectively), inter-test agreement was “very good” (*κ* = 0.97), and overall percent agreement was 99%. Two specimen pairs (both mucoid, one 13 days transport, one 1 day transport) had discordant Xpert results.

**Conclusion:**

OM-S-treated sputum specimens can undergo multi-day ambient-temperature storage as well as transport and yield Xpert results comparable to those of cold-chain-transported samples in Malawi.

## Background

Worldwide, tuberculosis (TB) is one of the top 10 causes of death, and the leading cause of mortality from a single infectious agent [1]. Likewise, it is a major public health concern and cause of mortality in Malawi. The country is also among the top 30 high-burden countries for TB/HIV co-infection. Case notifications for TB peaked at 28,000 in 2003, but detection has trended downward since then [2]. According to WHO’s 2017 report, Malawi’s case notification was 16, 853 cases per year [3]. The country’s overall TB cure rate during the past decade has been among the best in Africa, and reached 88% in 2010 [4]; however, case notification has remained virtually unchanged for many years. This has been attributed to several issues, including low sensitivity of smear microscopy and high prevalence of HIV, which is associated with high numbers of negative sputum smears [5].

Difficulties involved in the collection, storage and transportation of sputum samples to diagnostic centers is rampant and significantly affects the overall TB case detection effort. TB diagnostic laboratories usually necessitate to either process the specimen shortly after collection or store it at very low temperature to inhibit the growth of contaminating micro-organisms. The latter procedure entails additional labor and logistics costs for the processing and conservation of specimens, and reductions in sensitivity. To that end, pre-analytical technologies that preserve samples at ambient temperature storage and transport can increase TB detection. Program networks can benefit from such tools that maintain sample integrity, reduce culture contamination, and ease transport logistics. TB programs in developing countries where sputum sample collection sites are far from diagnostic centers need specimen storage and transport methods that function seamlessly at ambient temperature.

OMNIgene®•SPUTUM is supposed to serve as conducive medium for storage and transport of sputum samples at ambient-temperature and offers the flexibility of sample batching and reduced laboratory work. OMNIgene®•SPUTUM liquefies and decontaminates sputum, thus eliminating the NaOH/NALC processing step, and preserves MTb viability for at least 8 days at temperatures up to 40°C^6^. OMNIgene®•SPUTUM treated specimens are directly compatible with the full range of TB assays, including smear microscopy, liquid culture (BACTEC™ MGIT™ 960 System), solid culture, the Cepheid® GeneXpert® System, and other molecular tests [6–8]. In this study, Project HOPE collaborated with Malawi’s National TB Program (NTP) to field-test whether sputum specimens transported/stored in OMNIgene®•SPUTUM (OM-S) at ambient temperature are comparable to cold-chain-transported (Standard of Care; SOC) specimens for Xpert® MTB/RIF (Xpert) testing.

## Methods

Project HOPE’s TB REACH program has been operating in all three regions of Malawi (south, central and north) and comprises 14 implementation districts. Eight of those districts were in the southern region with a combined population of more than three million and more than a quarter of Malawi’s TB cases [5]. The six remaining implementation districts were in the central and northern regions of Malawi.

The data collection of this comparative study was completed in April 2016, and prior ethical clearance was granted by the Malawi National Health Sciences Research Committee. Sputum samples were collected at health facilities in seven implementation districts proportionally selected across the three regions (four in the south, two central and two northern).

### Patients and sample collection

Patients enrolled in the study were those eligible for Xpert screening per Malawi’s NTP guideline during the study period: *i*) HIV-positive outpatients who had been admitted to the ward with TB symptoms; *ii*) patients identified as having TB symptoms during screening at inpatient or in special clinics of the health facility; *iii*) patients with TB symptoms who were from countries with high multi-drug-resistant TB (MDR-TB) prevalence. Since some or most of the study participants or care givers may not have the literacy level to read and provide written consent, informed verbal consent was sought from all study participants for consistency purpose.

A maximum sample size of 377 was calculated based on 5% margin of error and 95% confidence interval. Sample collection was performed at 29 health facility sites within the seven selected districts. Each participating facility tracked its specimen collections until it reached an established quota. Participants were advised on how to produce quality sputum specimens. No patient was assisted by a respiratory therapy technician or stimulated with hypertonic saline aerosol to produce a sample. Two sputum specimens were collected per patient. To minimize frequency of visits and mitigate potential dropouts, samples were collected within one hour on the same day at most sites (i.e. both “spot” samples). However, some health facilities collected a spot sample followed by a “morning” sample the next day, as per Malawi’s NTP guideline, and some patients’ samples were both morning samples. Sputum character (e.g., mucoid, mucopurulent) was recorded for each participant. Each individual’s samples were randomly labeled Specimen #1 (standard-of-care group, or SOC) or Specimen #2 (OM-S group), and subjected to different treatments and procedures.

### Treatments

- **Specimen #1 (SOC group):** Each control specimen was stored and transported in a standard cold-chain system (2–8 °C), and was shipped to the district’s designated Xpert site (see details below) as soon as possible.
- **Specimen #2 (OM-S group):** Each intervention specimen was mixed with an equal amount of OMNIgene®•SPUTUM (i.e., 1:1 volume), re-capped tightly, and then stored and transported at ambient temperature (14–28 °C). These samples were also shipped to the designated Xpert site as soon as possible.

### Smear microscopy and Xpert testing

Per Malawi’s Xpert testing algorithm, not all sputum specimens must be evaluated by smear microscopy; those from HIV positive suspects, person from high MDR-TB burden countries and contacts, critically sick patients in the inpatient or special clinics including re-treatment cases are eligible for direct Xpert testing. As such, most patients in the study qualified for direct Xpert testing. If smear microscopy was performed, this was done at the collection facility on the day of sputum collection and the technique varied by site: Ziehl-Neelsen (14%) and fluorescence microscopy (11%). Smear results were recorded as positive or negative. In each study district, one central hospital with a functioning GeneXpert machine was designated as the district’s Xpert site. All specimens for each district were tested in the same GeneXpert machine, and any machine errors and codes were recorded. Xpert testing methods for each group are described below.

- **Specimen #1 (SOC group) Xpert testing:** The cold-chain-transported specimens were processed according to Cepheid’s Xpert protocol for expectorated sputum [9]. Briefly, each Xpert MTB/RIF cartridge was labeled with the specimen ID. A sterile transfer pipette was used to transfer approximately 1 mL of raw sputum to a conical, screw-capped tube. Alternatively, the entire specimen was processed in its original sputum collection container. A separate sterile pipette was used to add 2x volume of the manufacturer’s Sample Reagent (SR buffer) to the sputum (i.e., 2:1 volume ratio). The cap/lid was then re-secured and the mixture was either shaken vigorously (10–20 times) or vortexed for 30 s. The tube/container was rested upright for 5 min at room temperature, then shaken vigorously again (10–20 times) or vortexed for 30 s, and finally rested for another 10 min at room temperature. Each specimen was inspected to ensure liquefaction (i.e., no visible sputum clumps). Any sample not fully liquefied was shaken/vortexed and rested repeatedly until liquefaction was complete.
- **Specimen #2 (OM-S group) Xpert testing:** The OM-S-treated samples were also assayed directly (no additional processing) per Cepheid’s Xpert protocol for expectorated sputum [9]. Each specimen was mixed with 2x volume SR buffer (i.e., 4 mL of SR buffer was added to a typical 2 mL OM-S-treated sample). The mixture was then shaken/vortexed, incubated, and assessed for liquefaction as described for Specimen #1.

### Analysis and clinical results

All data from collection facilities and Xpert sites were entered into statistical software (SPSS v.20) and each specimen was assigned a unique identifier to link the data. Descriptive statistics were calculated and the groups’ TB case detection rates were compared. Transport/storage time was calculated as the interval between sputum collection and the Xpert assay date. Inter-test agreement was analyzed to derive Cohen’s *kappa* coefficient (*κ*) and percent-agreement values. The resultant *κ* value was assigned to a “strength of agreement” category [10]: 0.01–0.20 poor; 0.21–0.40 fair; 0.41–0.60 moderate; 0.61–0.80 substantial; 0.81–1.00 very good.

Cases were clinically managed according to Malawi NTP guidelines. There were two types of discordant test outcomes: *i*) OM-S-negative and SOC-positive, in which case the patient was considered clinically positive, and *ii*) OM-S-positive and SOC-negative, in which case the patient produced a new sputum sample for separate confirmatory testing. Only original Xpert results (not confirmatory results) were analyzed in the study.

## Results

In total, 362 specimen pairs were collected (96% of target sample size). Of these, 49 (13.5%) were excluded from analysis because of incomplete or miscoded patient/specimen ID or Xpert data. The study followed strict inclusion and exclusion criteria. All samples with less than 2 mL of solution were excluded. Table 1 summarizes the main demographic and clinical findings for the 313 participants.

**Table 1:** Patient demographic and clinical characteristics

	Total n	Result	
Age (yrs)	313	Median	42
		Mean ± SD	42.3 ± 13.5
		Range	2–79
Sex n (%)	312	Males	122 (39%)
		Females	191 (61%)
Region of Malawi	313	Southern	169 (54%)
		Central	85 (27%)
		Northern	59 (19%)
HIV status n (%)	312	Pos	250 (80%)
		Neg	31 (10%)
		Unknown	31 (10%)
Prior TB diagnosis n (%)	313	Yes	21 (9%)
		No	284 (91%)
Prior TB treatment n (%)	311	Yes	31 (10%)
		No	280 (90%)
Collection type			
Spot sample n (%)	313	Yes	245 (78%)
		No	68 (22%)
Morning sample n (%)	312	Yes	74 (24%)
		No	238 (76%)
Both types collected n (%)	312	Yes	21 (7%)
		No	291 (93%)
Sputum character n (%)	305	Mucoid	130 (43%)
		Purulent	28 (9%)
		Mucopurulent	44 (14%)
		Bloody	4 (1%)
		Saliva	41 (13%)
		Other	58 (19%)
Transport/storage time (days)	304	Median	6
		Mean ± SD	6.7 ± 5.0
		Range	0–29

*HIV* human immunodeficiency virus, *TB* tuberculosis. Results of interest agreement analysis of the use of utilizing Standard-of Care (SOC) Xpert had a positive agreement of 97% (*κ* = 0.97)

Mean patient age was 42.3 ± 13.5 years (range, 2–79 years), most participants (61%) were female, and the large majority (80%) were HIV-positive. Roughly 10% had had a prior TB diagnosis or had been treated for TB. One hundred sixty-nine patients (54%) were from districts in southern region, 85 (27%) were from the central region, and 59 (19%) were from the north. Most patients provided one or both samples as spot specimens (78%), 24% provided one or both as morning specimens, and only 7% had spot/morning combination. Of the 305 samples with sputum characteristics recorded, most (43%) were mucoid, 19% were non-specific character, 14% were mucopurulent, and 13% were classified as saliva.

Regarding the Xpert results, 37 (11.8%) of the OM-S samples were MTB-positive and 269 (85.9%) were MTB-negative; the corresponding results for the SOC samples were 35 (11.2%) MTB-positive and 270 (86.3%) MTB-negative. All MTB-positive samples were also rifampicin-sensitive. Only two specimens had discordant MTB findings: Specimen PH037 (mucoid, transport time 13 days, not smear-tested) and Specimen PH268 (mucoid, transport time 1 day, not smear-tested) were both OM-S-positive and SOC-negative. Smear testing status/information was recorded for 309 patients; however, the majority (77%) were not smear-tested and only six patients (8.6% of the 70 examined) were smear-positive. In four of these cases, the SOC and OM-S Xpert results were also both positive; in the two other smear-positive cases (Specimens PH294 and PH297; sputum character mucopurulent; transport/storage time 4 days), the SOC and OM-S Xpert results were both MTB-negative. Of the 64 patients that were smear-negative, only two (Specimens PH250 and PH255; saliva and mucopurulent, respectively; transport/storage 5 days) had Xpert results that disagreed with smear. In each case, the SOC and OM-S results were both MTB-positive. Statistical analysis revealed “very good” strength of agreement (*κ* = 0.97) between the OM-S and SOC Xpert tests, and 99% overall agreement of these results.

Eight SOC samples (2.6%) and seven OM-S samples (2.2%) had machine run error issues. These occurred in 10 specimen pairs: PH005, PH010, PH012, PH042, PH154, PH162, PH215, PH239, PH240, and PH290. Transport/storage time for these pairs ranged from 1 to 10 days, with seven at ≤6 days. Eight of the 10 specimens were saliva or non-specific sputum character, and four of those eight specimen pairs had “error” or “invalid” outputs for both the SOC and OM-S specimen; one SOC specimen (PH042) had “no result”. The remaining two of the 10 specimens were purulent (SOC MTB-negative, OM-S Error) and mucoid (SOC Error, OM-S MTB-negative), respectively. Error codes 5007 and 2127 were recorded; some codes were not specified. According to the recorded data, no errors/issues were directly caused by the treatment methods.

Regarding transport/storage time analyses, nine of the 313 specimen pairs with Xpert results had to be excluded due to inconsistent or missing data. Mean transport/storage time for the 304 analyzed pairs was 6.7 ± 5 days (median, 6 days; range, 0–29 days) (Table 1). Approximately 50% of the specimen pairs were Xpert-tested within 5 days of collection, 36% between 6 and 10 days, and 15% at 11 days or later (Table 2).

**Table 2:** Xpert and machine issue/error results listed by transport/storage time category

		MTB + n (% total n)			Machine Run / Error Issues	
Transport / Storage Time	Total n (%)	SOC	OM-S	Xpert Discrep^a^	SOC	OM-S
0–5 days	148 (49%)	15 (10%)	16 (11%)	1	2	3
6–10 days	110 (36%)	10 (9%)	10 (9%)	0	6	4
11–15 days	24 (8%)	5 (21%)	6 (25%)	1	0	0
16–29 days	22 (7%)	3 (14%)	3 (14%)	0	0	0
TOTALS^a^	**304 (100%)**	**33 (11%)**	**35 (12%)**	**2**	**8**	**7**

*Discrep* discrepancy of Xpert results (both SOC sample MTB-neg, OM-S sample MTB-pos), *MTB Mycobacterium tuberculosis*, *OM-S* OMNIgene®•SPUTUM, *SOC* standard of care

## Discussion

Xpert TB detection rates were comparable in the SOC and OM-S groups (both approximately 11% positivity) even though 80% of patients were HIV-positive and sputum from such individuals often contains low numbers of bacilli. Our findings suggest that sputum transported/stored in OM-S was compatible with Xpert and does not affect the sensitivity of the assay, even when a high proportion of samples were low-positive. The Xpert results were concordant with available smear results, and statistical analysis revealed 99% overall agreement between the OM-S and SOC Xpert results, as well as 0.97 Cohen’s Kappa Index (*κ)* value signaling strongest category of inter-test agreement [10].

The two specimens with discordant Xpert results were mucoid with transport/storage duration of 1 and 13 days. In both cases, the discordance could be due to low-positive TB status; however, this can only be speculated without supportive smear data. Machine run issues/errors were comparable in the two groups, and most were associated with sputum samples characterized as saliva or non-specific nature. As observed in another evaluation of OM-S transport to Xpert testing in Nepal [11], the Xpert error codes reported in this study revealed no cartridge issues that suggest reagent incompatibility with the Xpert system. The two error codes specified indicated issues independent of the sample treatment method.

The mean transport/storage time for specimens was 6.7 days and approximately 85% were Xpert-tested between 1 and 10 days post-collection; however, almost 50 samples were tested between 11 and 29 days. The results indicate that sputum transport or batching in OM-S for two weeks or more may not interfere with the Xpert assay.

## Conclusions

Sputum samples can be collected at remote locations, transported/stored with OMNIgene®•SPUTUM as a medium at ambient temperature for 7–8 days, and then successfully Xpert-tested at hub sites using Cepheid’s expectorated sputum protocol [9]. Statistical analysis indicate that OMNIgene®•SPUTUM treated samples (OM-S) perform comparably to cold-chain-transported samples in this protocol.

Treating raw sputum samples with OMNIgene®•SPUTUM as storage and transportation medium can potentially help TB programs diagnostic endeavors through: *i*) shipping/storage at ambient temperature from remote collection sites to GeneXpert diagnostic centers, *ii*) eliminating additional time needed for decontamination steps in the laboratory, and *iii*) a larger time window for sputum testing. This study recommends broader investigations of OM-S as a sputum transport medium for culture, microscopy and other types of TB diagnostics.

## Abbreviations

HIV: Human immunodeficiency virus
IRB: Institutional review board
MDR-TB: Multi drug resistant TB
MOH: Ministry of Health
MTB: *Mycobacterium tuberculosis*
NHSRC: National Health Science Research Committee
NTP: National TB Program
OM-S: OMNIgene Sample
SOC: Standard of Care
SPSS: Statistical Package for Social Sciences
SR: Sample Reagent
TB: Tuberculosis
WHO: World Health Organization
